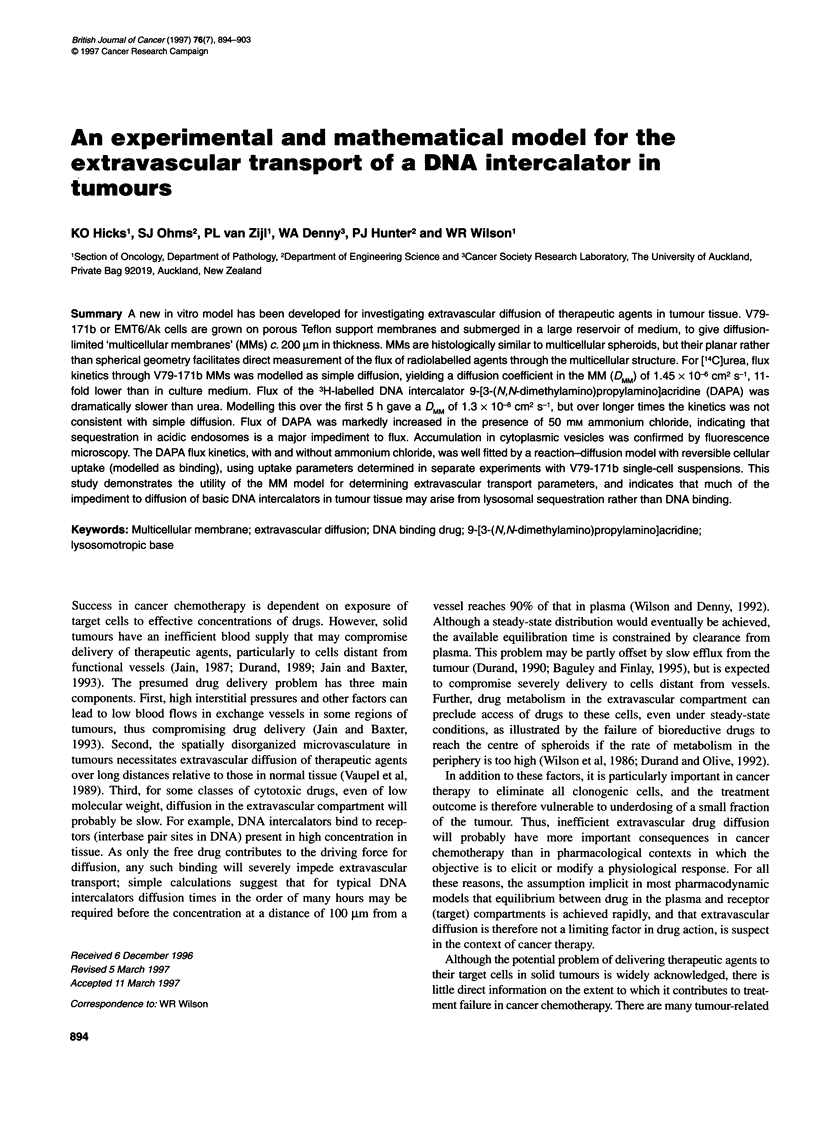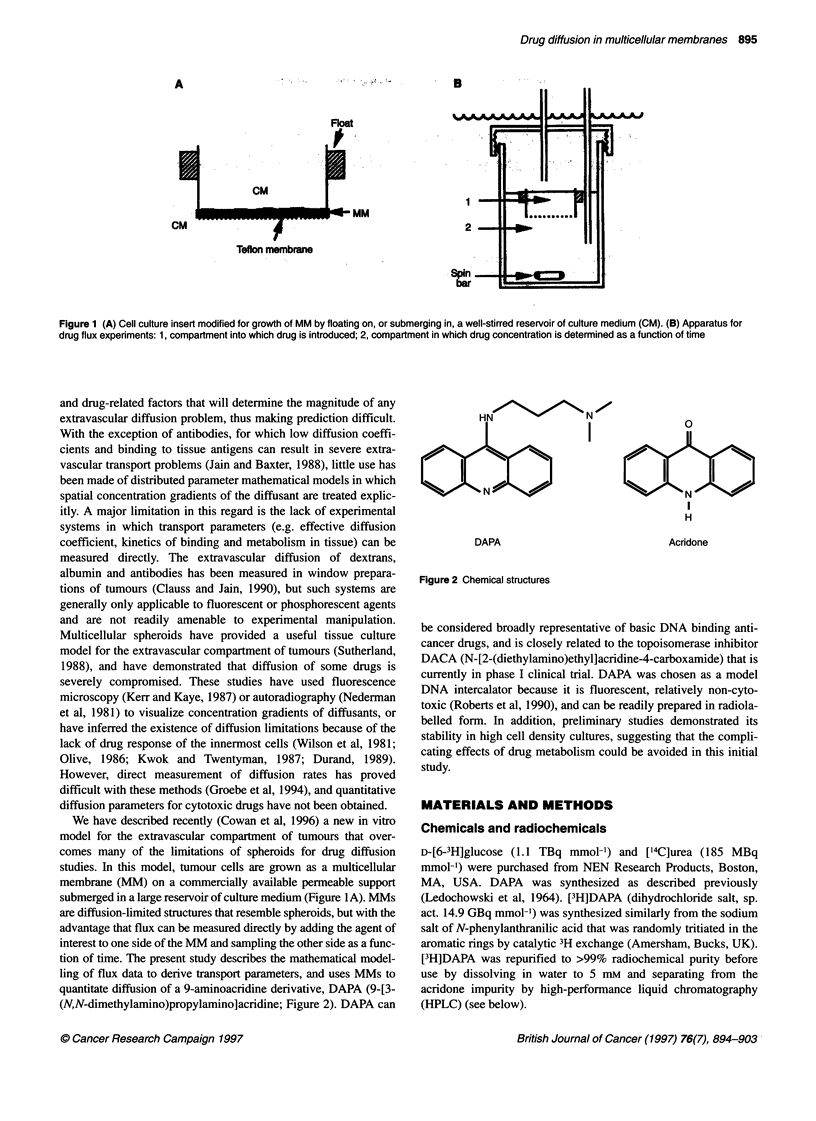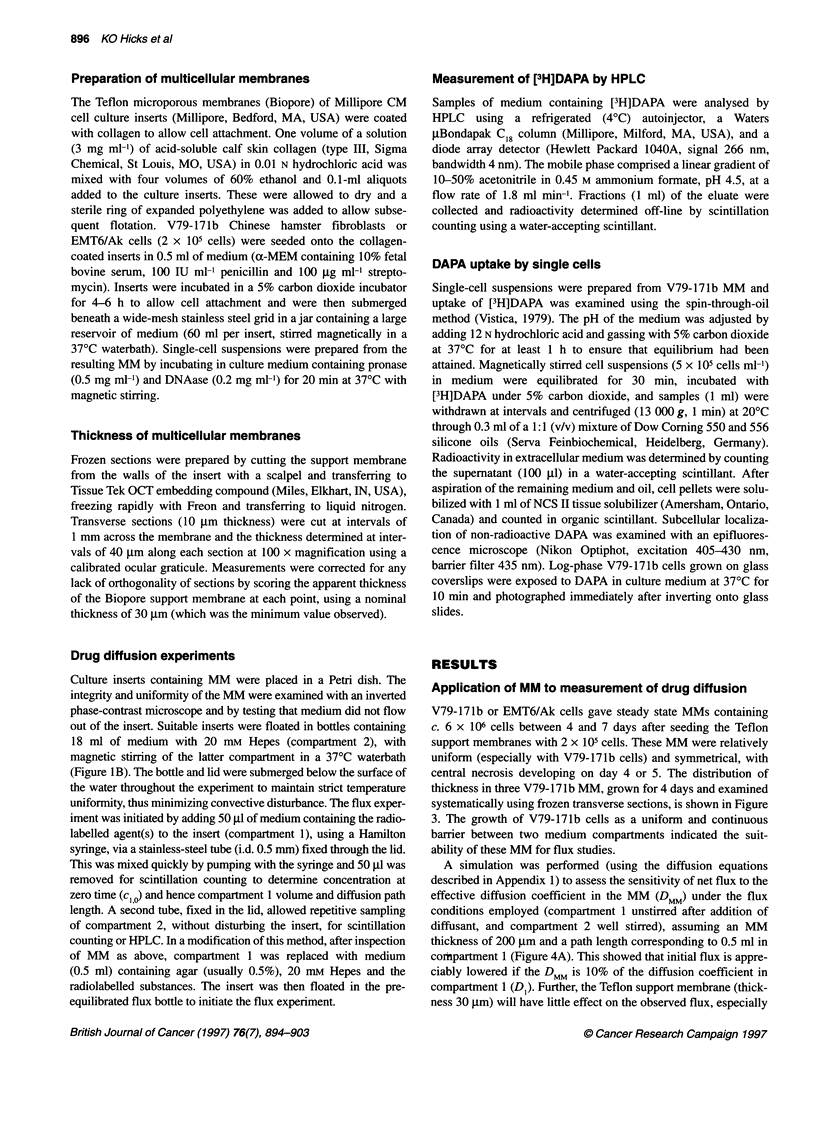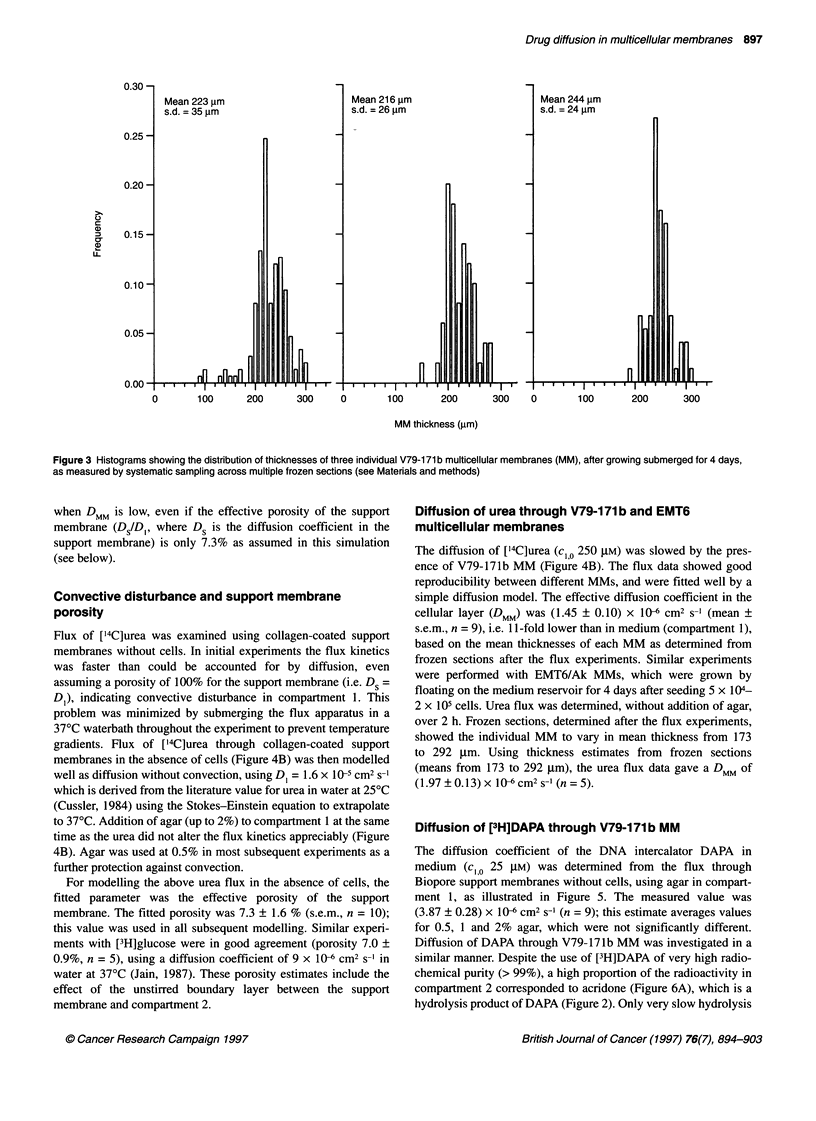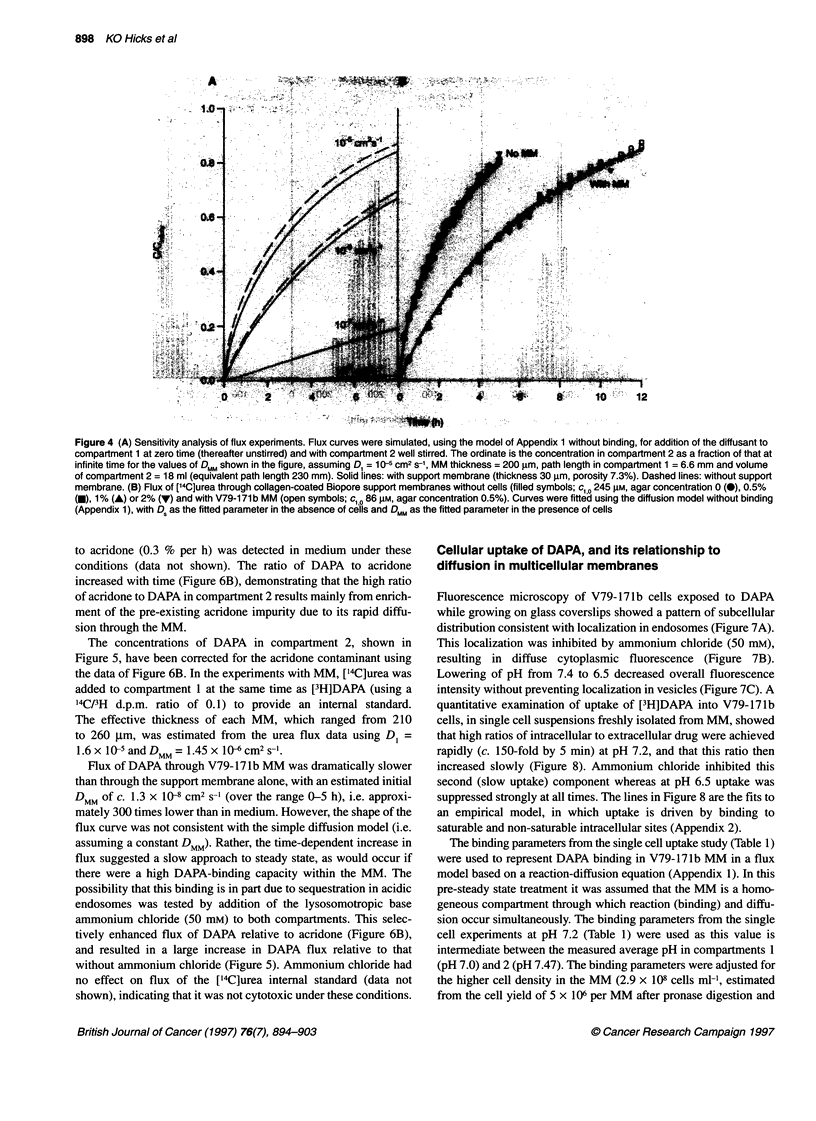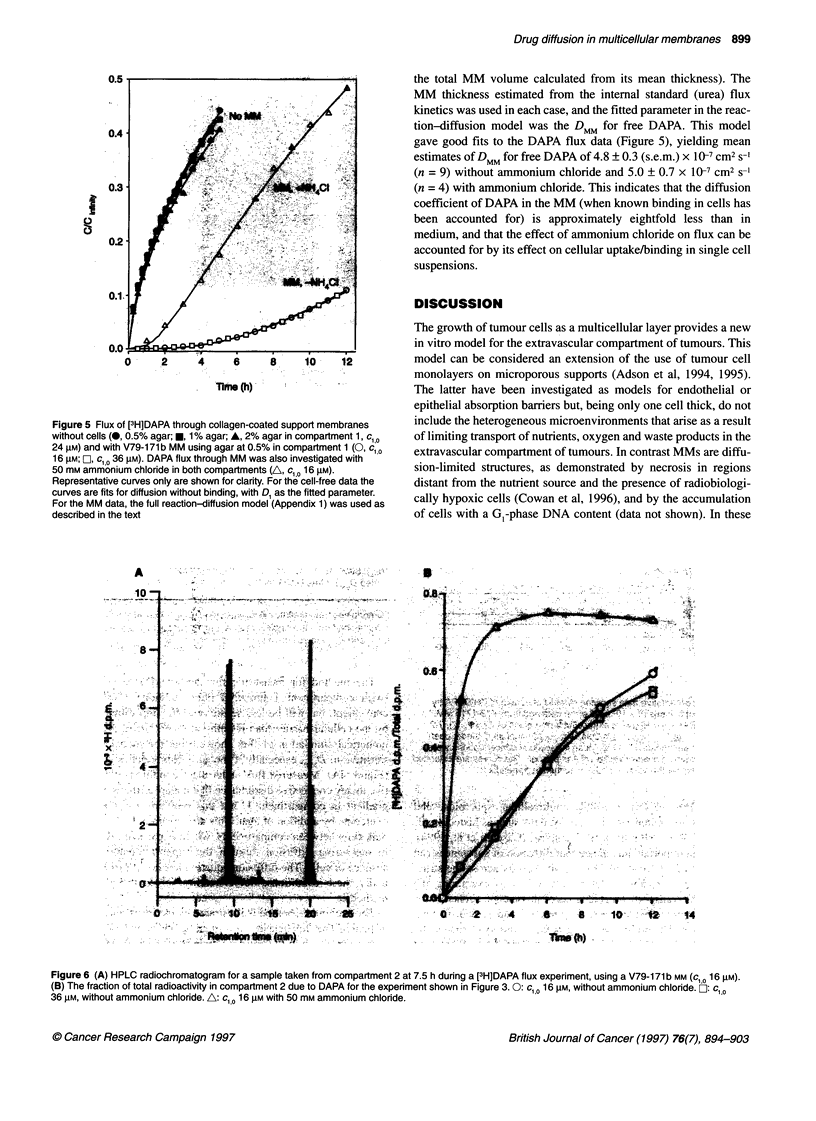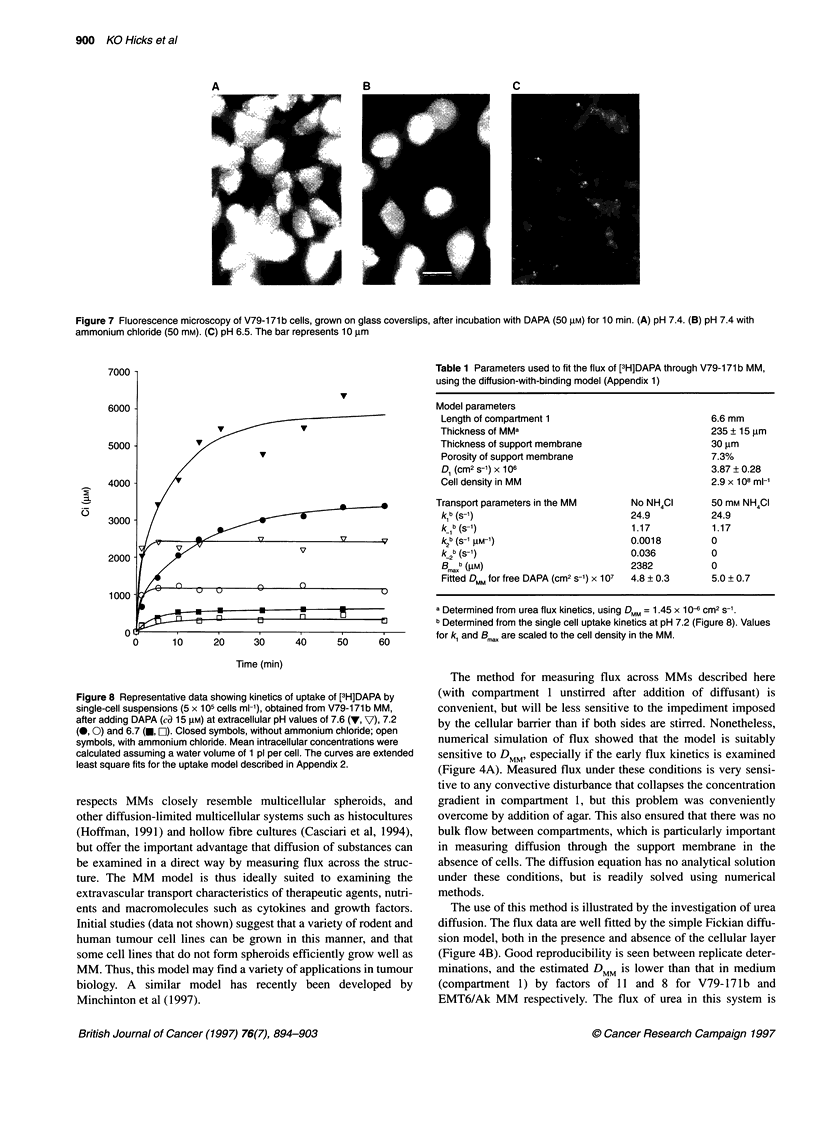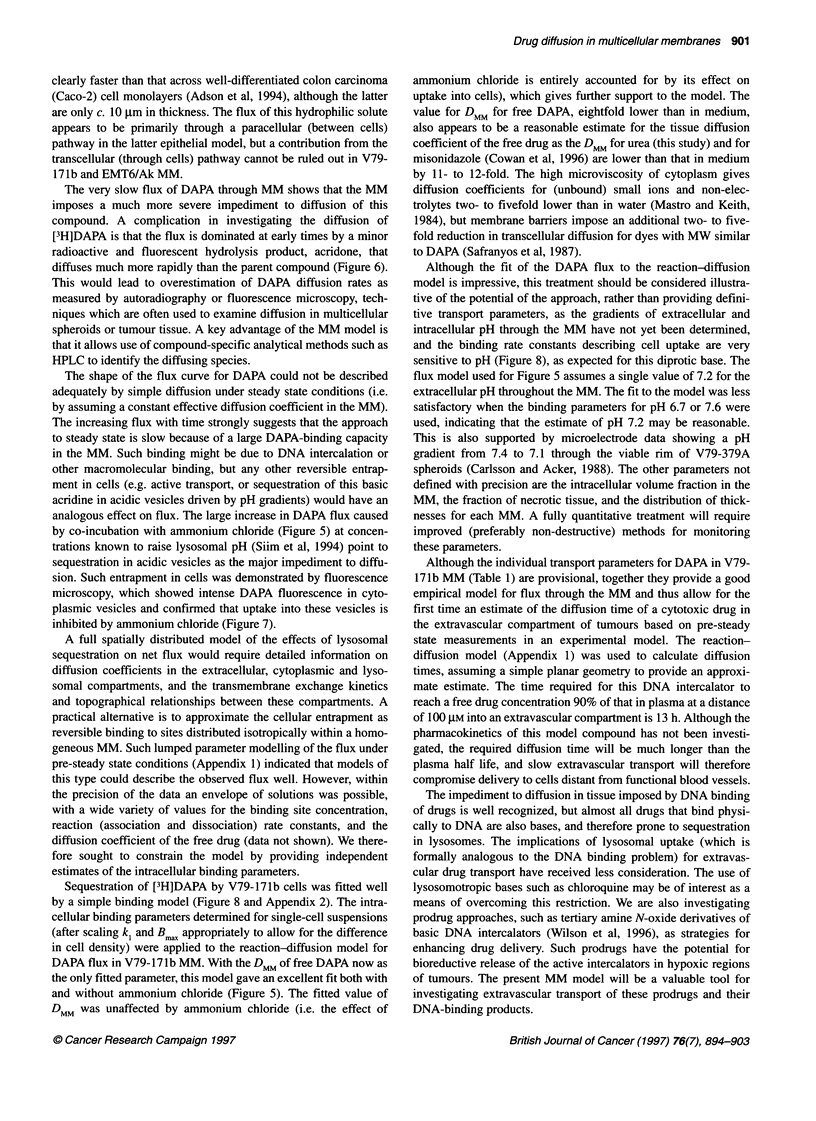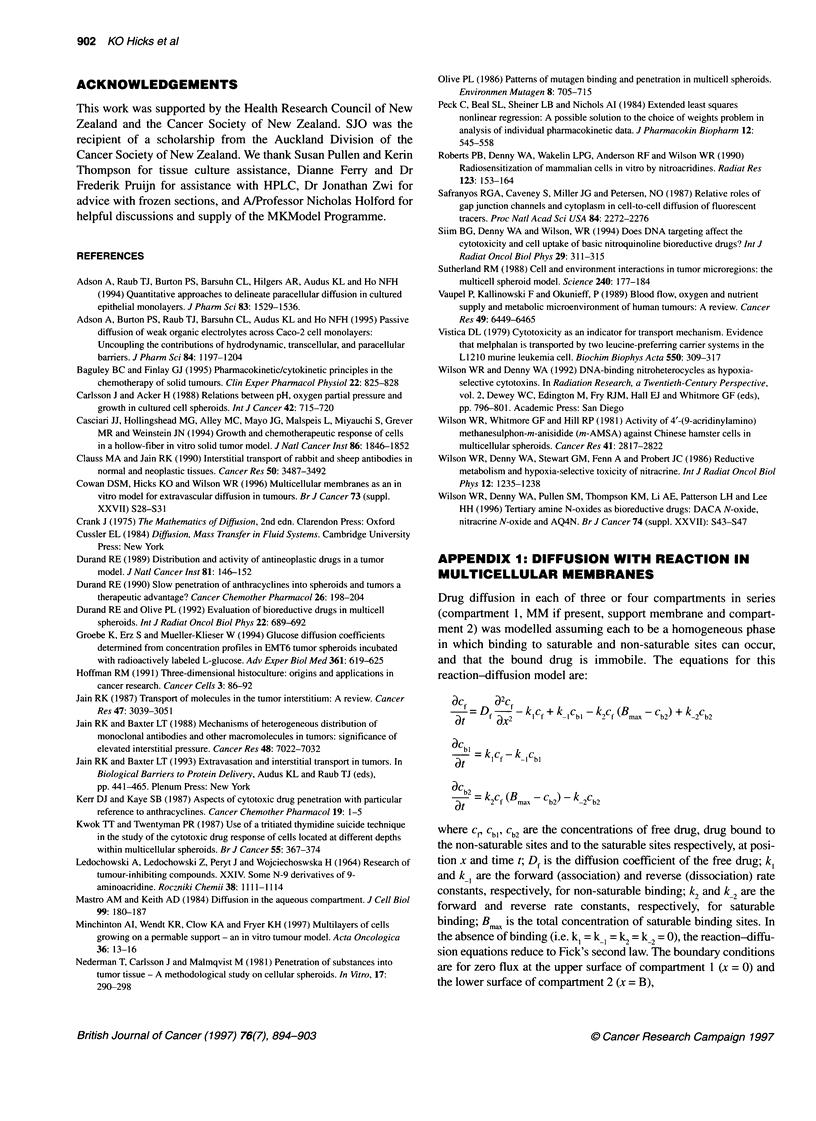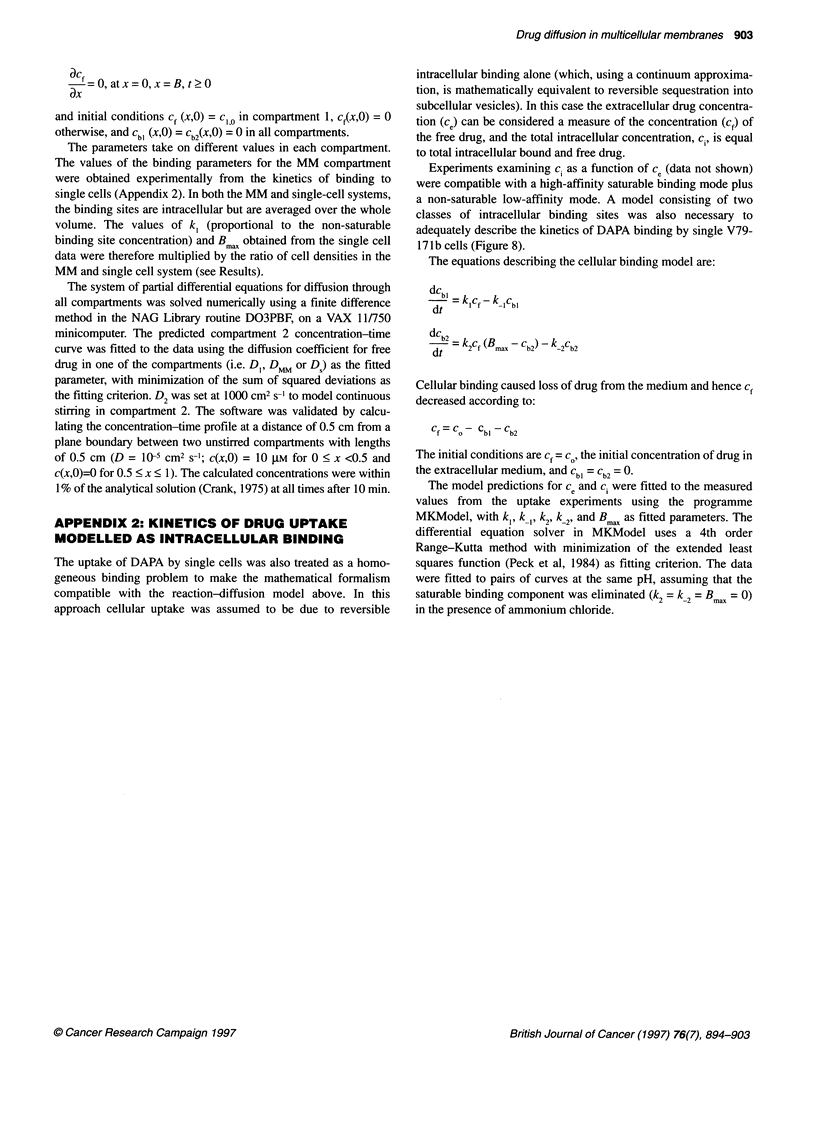# An experimental and mathematical model for the extravascular transport of a DNA intercalator in tumours.

**DOI:** 10.1038/bjc.1997.481

**Published:** 1997

**Authors:** K. O. Hicks, S. J. Ohms, P. L. van Zijl, W. A. Denny, P. J. Hunter, W. R. Wilson

**Affiliations:** Department of Pathology, The University of Auckland, New Zealand.

## Abstract

**Images:**


British Journal of Cancer (1997) 76(7), 894–903
© 1997 Cancer Research Campaign

### An experimental and mathematical model for the extravascular transport of a DNA intercalator in tumours

**KO Hicks[1], SJ Ohms[2], PL van Zijl[1], WA Denny[3], PJ Hunter[2] and WR Wilson[1]**

[1]Section of Oncology, Department of Pathology, [2]Department of Engineering Science and [3]Cancer Society Research Laboratory, The University of Auckland, Private Bag 92019, Auckland, New Zealand

**Summary** A new in vitro model has been developed for investigating extravascular diffusion of therapeutic agents in tumour tissue. V79-171b or EMT6/Ak cells are grown on porous Teflon support membranes and submerged in a large reservoir of medium, to give diffusion-limited 'multicellular membranes' (MMs) *c.* 200 μm in thickness. MMs are histologically similar to multicellular spheroids, but their planar rather than spherical geometry facilitates direct measurement of the flux of radiolabelled agents through the multicellular structure. For [$^{14}$C]urea, flux kinetics through V79-171b MMs was modelled as simple diffusion, yielding a diffusion coefficient in the MM ($D_{MM}$) of $1.45 \times 10^{-6}$ cm$^2$ s$^{-1}$, 11-fold lower than in culture medium. Flux of the $^3$H-labelled DNA intercalator 9-[3-(*N,N*-dimethylamino)propylamino]acridine (DAPA) was dramatically slower than urea. Modelling this over the first 5 h gave a $D_{MM}$ of $1.3 \times 10^{-8}$ cm$^2$ s$^{-1}$, but over longer times the kinetics was not consistent with simple diffusion. Flux of DAPA was markedly increased in the presence of 50 mM ammonium chloride, indicating that sequestration in acidic endosomes is a major impediment to flux. Accumulation in cytoplasmic vesicles was confirmed by fluorescence microscopy. The DAPA flux kinetics, with and without ammonium chloride, was well fitted by a reaction–diffusion model with reversible cellular uptake (modelled as binding), using uptake parameters determined in separate experiments with V79-171b single-cell suspensions. This study demonstrates the utility of the MM model for determining extravascular transport parameters, and indicates that much of the impediment to diffusion of basic DNA intercalators in tumour tissue may arise from lysosomal sequestration rather than DNA binding.

**Keywords:** Multicellular membrane; extravascular diffusion; DNA binding drug; 9-[3-(*N,N*-dimethylamino)propylamino]acridine; lysosomotropic base

Success in cancer chemotherapy is dependent on exposure of target cells to effective concentrations of drugs. However, solid tumours have an inefficient blood supply that may compromise delivery of therapeutic agents, particularly to cells distant from functional vessels (Jain, 1987; Durand, 1989; Jain and Baxter, 1993). The presumed drug delivery problem has three main components. First, high interstitial pressures and other factors can lead to low blood flows in exchange vessels in some regions of tumours, thus compromising drug delivery (Jain and Baxter, 1993). Second, the spatially disorganized microvasculature in tumours necessitates extravascular diffusion of therapeutic agents over long distances relative to those in normal tissue (Vaupel et al, 1989). Third, for some classes of cytotoxic drugs, even of low molecular weight, diffusion in the extravascular compartment will probably be slow. For example, DNA intercalators bind to receptors (interbase pair sites in DNA) present in high concentration in tissue. As only the free drug contributes to the driving force for diffusion, any such binding will severely impede extravascular transport; simple calculations suggest that for typical DNA intercalators diffusion times in the order of many hours may be required before the concentration at a distance of 100 μm from a vessel reaches 90% of that in plasma (Wilson and Denny, 1992). Although a steady-state distribution would eventually be achieved, the available equilibration time is constrained by clearance from plasma. This problem may be partly offset by slow efflux from the tumour (Durand, 1990; Baguley and Finlay, 1995), but is expected to compromise severely delivery to cells distant from vessels. Further, drug metabolism in the extravascular compartment can preclude access of drugs to these cells, even under steady-state conditions, as illustrated by the failure of bioreductive drugs to reach the centre of spheroids if the rate of metabolism in the periphery is too high (Wilson et al, 1986; Durand and Olive, 1992).

In addition to these factors, it is particularly important in cancer therapy to eliminate all clonogenic cells, and the treatment outcome is therefore vulnerable to underdosing of a small fraction of the tumour. Thus, inefficient extravascular drug diffusion will probably have more important consequences in cancer chemotherapy than in pharmacological contexts in which the objective is to elicit or modify a physiological response. For all these reasons, the assumption implicit in most pharmacodynamic models that equilibrium between drug in the plasma and receptor (target) compartments is achieved rapidly, and that extravascular diffusion is therefore not a limiting factor in drug action, is suspect in the context of cancer therapy.

Although the potential problem of delivering therapeutic agents to their target cells in solid tumours is widely acknowledged, there is little direct information on the extent to which it contributes to treatment failure in cancer chemotherapy. There are many tumour-related

*Received 6 December 1996*
*Revised 5 March 1997*
*Accepted 11 March 1997*

*Correspondence to:* WR Wilson

**Figure 1** (**A**) Cell culture insert modified for growth of MM by floating on, or submerging in, a well-stirred reservoir of culture medium (CM). (**B**) Apparatus for drug flux experiments: 1, compartment into which drug is introduced; 2, compartment in which drug concentration is determined as a function of time and drug-related factors that will determine the magnitude of any extravascular diffusion problem, thus making prediction difficult. With the exception of antibodies, for which low diffusion coefficients and binding to tissue antigens can result in severe extravascular transport problems (Jain and Baxter, 1988), little use has been made of distributed parameter mathematical models in which spatial concentration gradients of the diffusant are treated explicitly. A major limitation in this regard is the lack of experimental systems in which transport parameters (e.g. effective diffusion coefficient, kinetics of binding and metabolism in tissue) can be measured directly. The extravascular diffusion of dextrans, albumin and antibodies has been measured in window preparations of tumours (Clauss and Jain, 1990), but such systems are generally only applicable to fluorescent or phosphorescent agents and are not readily amenable to experimental manipulation. Multicellular spheroids have provided a useful tissue culture model for the extravascular compartment of tumours (Sutherland, 1988), and have demonstrated that diffusion of some drugs is severely compromised. These studies have used fluorescence microscopy (Kerr and Kaye, 1987) or autoradiography (Nederman et al, 1981) to visualize concentration gradients of diffusants, or have inferred the existence of diffusion limitations because of the lack of drug response of the innermost cells (Wilson et al, 1981; Olive, 1986; Kwok and Twentyman, 1987; Durand, 1989). However, direct measurement of diffusion rates has proved difficult with these methods (Groebe et al, 1994), and quantitative diffusion parameters for cytotoxic drugs have not been obtained.

We have described recently (Cowan et al, 1996) a new in vitro model for the extravascular compartment of tumours that overcomes many of the limitations of spheroids for drug diffusion studies. In this model, tumour cells are grown as a multicellular membrane (MM) on a commercially available permeable support submerged in a large reservoir of culture medium (Figure 1A). MMs are diffusion-limited structures that resemble spheroids, but with the advantage that flux can be measured directly by adding the agent of interest to one side of the MM and sampling the other side as a function of time. The present study describes the mathematical modelling of flux data to derive transport parameters, and uses MMs to quantitate diffusion of a 9-aminoacridine derivative, DAPA (9-[3-(*N*,*N*-dimethylamino)propylamino]acridine; Figure 2). DAPA can

DAPA Acridone

**Figure 2** Chemical structures be considered broadly representative of basic DNA binding anticancer drugs, and is closely related to the topoisomerase inhibitor DACA (N-[2-(diethylamino)ethyl]acridine-4-carboxamide) that is currently in phase I clinical trial. DAPA was chosen as a model DNA intercalator because it is fluorescent, relatively non-cytotoxic (Roberts et al, 1990), and can be readily prepared in radiolabelled form. In addition, preliminary studies demonstrated its stability in high cell density cultures, suggesting that the complicating effects of drug metabolism could be avoided in this initial study.

### MATERIALS AND METHODS

### Chemicals and radiochemicals

D-[6-$^3$H]glucose (1.1 TBq mmol$^{-1}$) and [$^{14}$C]urea (185 MBq mmol$^{-1}$) were purchased from NEN Research Products, Boston, MA, USA. DAPA was synthesized as described previously (Ledochowski et al, 1964). [$^3$H]DAPA (dihydrochloride salt, sp. act. 14.9 GBq mmol$^{-1}$) was synthesized similarly from the sodium salt of *N*-phenylanthranilic acid that was randomly tritiated in the aromatic rings by catalytic $^3$H exchange (Amersham, Bucks, UK). [$^3$H]DAPA was repurified to >99% radiochemical purity before use by dissolving in water to 5 mM and separating from the acridone impurity by high-performance liquid chromatography (HPLC) (see below).

© Cancer Research Campaign 1997 

### Preparation of multicellular membranes

The Teflon microporous membranes (Biopore) of Millipore CM cell culture inserts (Millipore, Bedford, MA, USA) were coated with collagen to allow cell attachment. One volume of a solution (3 mg $ml^{-1}$) of acid-soluble calf skin collagen (type III, Sigma Chemical, St Louis, MO, USA) in 0.01 N hydrochloric acid was mixed with four volumes of 60% ethanol and 0.1-ml aliquots added to the culture inserts. These were allowed to dry and a sterile ring of expanded polyethylene was added to allow subsequent flotation. V79-171b Chinese hamster fibroblasts or EMT6/Ak cells ($2 \times 10^5$ cells) were seeded onto the collagen-coated inserts in 0.5 ml of medium (α-MEM containing 10% fetal bovine serum, 100 IU $ml^{-1}$ penicillin and 100 μg $ml^{-1}$ streptomycin). Inserts were incubated in a 5% carbon dioxide incubator for 4–6 h to allow cell attachment and were then submerged beneath a wide-mesh stainless steel grid in a jar containing a large reservoir of medium (60 ml per insert, stirred magnetically in a 37°C waterbath). Single-cell suspensions were prepared from the resulting MM by incubating in culture medium containing pronase (0.5 mg $ml^{-1}$) and DNAase (0.2 mg $ml^{-1}$) for 20 min at 37°C with magnetic stirring.

### Thickness of multicellular membranes

Frozen sections were prepared by cutting the support membrane from the walls of the insert with a scalpel and transferring to Tissue Tek OCT embedding compound (Miles, Elkhart, IN, USA), freezing rapidly with Freon and transferring to liquid nitrogen. Transverse sections (10 μm thickness) were cut at intervals of 1 mm across the membrane and the thickness determined at intervals of 40 μm along each section at 100 × magnification using a calibrated ocular graticule. Measurements were corrected for any lack of orthogonality of sections by scoring the apparent thickness of the Biopore support membrane at each point, using a nominal thickness of 30 μm (which was the minimum value observed).

### Drug diffusion experiments

Culture inserts containing MM were placed in a Petri dish. The integrity and uniformity of the MM were examined with an inverted phase-contrast microscope and by testing that medium did not flow out of the insert. Suitable inserts were floated in bottles containing 18 ml of medium with 20 mM Hepes (compartment 2), with magnetic stirring of the latter compartment in a 37°C waterbath (Figure 1B). The bottle and lid were submerged below the surface of the water throughout the experiment to maintain strict temperature uniformity, thus minimizing convective disturbance. The flux experiment was initiated by adding 50 μl of medium containing the radiolabelled agent(s) to the insert (compartment 1), using a Hamilton syringe, via a stainless-steel tube (i.d. 0.5 mm) fixed through the lid. This was mixed quickly by pumping with the syringe and 50 μl was removed for scintillation counting to determine concentration at zero time ($c_{1,0}$) and hence compartment 1 volume and diffusion path length. A second tube, fixed in the lid, allowed repetitive sampling of compartment 2, without disturbing the insert, for scintillation counting or HPLC. In a modification of this method, after inspection of MM as above, compartment 1 was replaced with medium (0.5 ml) containing agar (usually 0.5%), 20 mM Hepes and the radiolabelled substances. The insert was then floated in the pre-equilibrated flux bottle to initiate the flux experiment.

### Measurement of [$^3$H]DAPA by HPLC

Samples of medium containing [$^3$H]DAPA were analysed by HPLC using a refrigerated (4°C) autoinjector, a Waters μBondapak $C_{18}$ column (Millipore, Milford, MA, USA), and a diode array detector (Hewlett Packard 1040A, signal 266 nm, bandwidth 4 nm). The mobile phase comprised a linear gradient of 10–50% acetonitrile in 0.45 M ammonium formate, pH 4.5, at a flow rate of 1.8 ml $min^{-1}$. Fractions (1 ml) of the eluate were collected and radioactivity determined off-line by scintillation counting using a water-accepting scintillant.

### DAPA uptake by single cells

Single-cell suspensions were prepared from V79-171b MM and uptake of [$^3$H]DAPA was examined using the spin-through-oil method (Vistica, 1979). The pH of the medium was adjusted by adding 12 N hydrochloric acid and gassing with 5% carbon dioxide at 37°C for at least 1 h to ensure that equilibrium had been attained. Magnetically stirred cell suspensions ($5 \times 10^5$ cells $ml^{-1}$) in medium were equilibrated for 30 min, incubated with [$^3$H]DAPA under 5% carbon dioxide, and samples (1 ml) were withdrawn at intervals and centrifuged (13 000 **g**, 1 min) at 20°C through 0.3 ml of a 1:1 (v/v) mixture of Dow Corning 550 and 556 silicone oils (Serva Feinbiochemical, Heidelberg, Germany). Radioactivity in extracellular medium was determined by counting the supernatant (100 μl) in a water-accepting scintillant. After aspiration of the remaining medium and oil, cell pellets were solubilized with 1 ml of NCS II tissue solubilizer (Amersham, Ontario, Canada) and counted in organic scintillant. Subcellular localization of non-radioactive DAPA was examined with an epifluorescence microscope (Nikon Optiphot, excitation 405–430 nm, barrier filter 435 nm). Log-phase V79-171b cells grown on glass coverslips were exposed to DAPA in culture medium at 37°C for 10 min and photographed immediately after inverting onto glass slides.

### RESULTS

### Application of MM to measurement of drug diffusion

V79-171b or EMT6/Ak cells gave steady state MMs containing *c.* $6 \times 10^6$ cells between 4 and 7 days after seeding the Teflon support membranes with $2 \times 10^5$ cells. These MM were relatively uniform (especially with V79-171b cells) and symmetrical, with central necrosis developing on day 4 or 5. The distribution of thickness in three V79-171b MM, grown for 4 days and examined systematically using frozen transverse sections, is shown in Figure 3. The growth of V79-171b cells as a uniform and continuous barrier between two medium compartments indicated the suitability of these MM for flux studies.

A simulation was performed (using the diffusion equations described in Appendix 1) to assess the sensitivity of net flux to the effective diffusion coefficient in the MM ($D_{MM}$) under the flux conditions employed (compartment 1 unstirred after addition of diffusant, and compartment 2 well stirred), assuming an MM thickness of 200 μm and a path length corresponding to 0.5 ml in compartment 1 (Figure 4A). This showed that initial flux is appreciably lowered if the $D_{MM}$ is 10% of the diffusion coefficient in compartment 1 ($D_1$). Further, the Teflon support membrane (thickness 30 μm) will have little effect on the observed flux, especially

© Cancer Research Campaign 1997

**Figure 3** Histograms showing the distribution of thicknesses of three individual V79-171b multicellular membranes (MM), after growing submerged for 4 days, as measured by systematic sampling across multiple frozen sections (see Materials and methods)

when $D_{MM}$ is low, even if the effective porosity of the support membrane ($D_S/D_1$, where $D_S$ is the diffusion coefficient in the support membrane) is only 7.3% as assumed in this simulation (see below).

### Convective disturbance and support membrane porosity

Flux of [$^{14}$C]urea was examined using collagen-coated support membranes without cells. In initial experiments the flux kinetics was faster than could be accounted for by diffusion, even assuming a porosity of 100% for the support membrane (i.e. $D_S = D_1$), indicating convective disturbance in compartment 1. This problem was minimized by submerging the flux apparatus in a 37°C waterbath throughout the experiment to prevent temperature gradients. Flux of [$^{14}$C]urea through collagen-coated support membranes in the absence of cells (Figure 4B) was then modelled well as diffusion without convection, using $D_1 = 1.6 \times 10^{-5}$ cm$^2$ s$^{-1}$ which is derived from the literature value for urea in water at 25°C (Cussler, 1984) using the Stokes–Einstein equation to extrapolate to 37°C. Addition of agar (up to 2%) to compartment 1 at the same time as the urea did not alter the flux kinetics appreciably (Figure 4B). Agar was used at 0.5% in most subsequent experiments as a further protection against convection.

For modelling the above urea flux in the absence of cells, the fitted parameter was the effective porosity of the support membrane. The fitted porosity was 7.3 ± 1.6 % (s.e.m., $n = 10$); this value was used in all subsequent modelling. Similar experiments with [$^3$H]glucose were in good agreement (porosity 7.0 ± 0.9%, $n = 5$), using a diffusion coefficient of $9 \times 10^{-6}$ cm$^2$ s$^{-1}$ in water at 37°C (Jain, 1987). These porosity estimates include the effect of the unstirred boundary layer between the support membrane and compartment 2.

### Diffusion of urea through V79-171b and EMT6 multicellular membranes

The diffusion of [$^{14}$C]urea ($c_{1,0}$ 250 μM) was slowed by the presence of V79-171b MM (Figure 4B). The flux data showed good reproducibility between different MMs, and were fitted well by a simple diffusion model. The effective diffusion coefficient in the cellular layer ($D_{MM}$) was $(1.45 \pm 0.10) \times 10^{-6}$ cm$^2$ s$^{-1}$ (mean ± s.e.m., $n = 9$), i.e. 11-fold lower than in medium (compartment 1), based on the mean thicknesses of each MM as determined from frozen sections after the flux experiments. Similar experiments were performed with EMT6/Ak MMs, which were grown by floating on the medium reservoir for 4 days after seeding $5 \times 10^4$–$2 \times 10^5$ cells. Urea flux was determined, without addition of agar, over 2 h. Frozen sections, determined after the flux experiments, showed the individual MM to vary in mean thickness from 173 to 292 μm. Using thickness estimates from frozen sections (means from 173 to 292 μm), the urea flux data gave a $D_{MM}$ of $(1.97 \pm 0.13) \times 10^{-6}$ cm$^2$ s$^{-1}$ ($n = 5$).

### Diffusion of [$^3$H]DAPA through V79-171b MM

The diffusion coefficient of the DNA intercalator DAPA in medium ($c_{1,0}$ 25 μM) was determined from the flux through Biopore support membranes without cells, using agar in compartment 1, as illustrated in Figure 5. The measured value was $(3.87 \pm 0.28) \times 10^{-6}$ cm$^2$ s$^{-1}$ ($n = 9$); this estimate averages values for 0.5, 1 and 2% agar, which were not significantly different. Diffusion of DAPA through V79-171b MM was investigated in a similar manner. Despite the use of [$^3$H]DAPA of very high radiochemical purity (> 99%), a high proportion of the radioactivity in compartment 2 corresponded to acridone (Figure 6A), which is a hydrolysis product of DAPA (Figure 2). Only very slow hydrolysis

**Figure 4** (**A**) Sensitivity analysis of flux experiments. Flux curves were simulated, using the model of Appendix 1 without binding, for addition of the diffusant to compartment 1 at zero time (thereafter unstirred) and with compartment 2 well stirred. The ordinate is the concentration in compartment 2 as a fraction of that at infinite time for the values of $D_{MM}$ shown in the figure, assuming $D_l$ = $10^{-5}$ cm$^2$ s$^{-1}$, MM thickness = 200 μm, path length in compartment 1 = 6.6 mm and volume of compartment 2 = 18 ml (equivalent path length 230 mm). Solid lines: with support membrane (thickness 30 μm, porosity 7.3%). Dashed lines: without support membrane. (**B**) Flux of [$^{14}$C]urea through collagen-coated Biopore support membranes without cells (filled symbols; $c_{1,0}$ 245 μM, agar concentration 0 (●), 0.5% (■), 1% (▲) or 2% (▼) and with V79-171b MM (open symbols; $c_{1,0}$ 86 μM, agar concentration 0.5%). Curves were fitted using the diffusion model without binding (Appendix 1), with $D_s$ as the fitted parameter in the absence of cells and $D_{MM}$ as the fitted parameter in the presence of cells to acridone (0.3 % per h) was detected in medium under these conditions (data not shown). The ratio of DAPA to acridone increased with time (Figure 6B), demonstrating that the high ratio of acridone to DAPA in compartment 2 results mainly from enrichment of the pre-existing acridone impurity due to its rapid diffusion through the MM.

The concentrations of DAPA in compartment 2, shown in Figure 5, have been corrected for the acridone contaminant using the data of Figure 6B. In the experiments with MM, [$^{14}$C]urea was added to compartment 1 at the same time as [$^3$H]DAPA (using a $^{14}$C/$^3$H d.p.m. ratio of 0.1) to provide an internal standard. The effective thickness of each MM, which ranged from 210 to 260 μm, was estimated from the urea flux data using $D_l$ = $1.6 \times 10^{-5}$ and $D_{MM}$ = $1.45 \times 10^{-6}$ cm$^2$ s$^{-1}$.

Flux of DAPA through V79-171b MM was dramatically slower than through the support membrane alone, with an estimated initial $D_{MM}$ of *c.* $1.3 \times 10^{-8}$ cm$^2$ s$^{-1}$ (over the range 0–5 h), i.e. approximately 300 times lower than in medium. However, the shape of the flux curve was not consistent with the simple diffusion model (i.e. assuming a constant $D_{MM}$). Rather, the time-dependent increase in flux suggested a slow approach to steady state, as would occur if there were a high DAPA-binding capacity within the MM. The possibility that this binding is in part due to sequestration in acidic endosomes was tested by addition of the lysosomotropic base ammonium chloride (50 mM) to both compartments. This selectively enhanced flux of DAPA relative to acridone (Figure 6B), and resulted in a large increase in DAPA flux relative to that without ammonium chloride (Figure 5). Ammonium chloride had no effect on flux of the [$^{14}$C]urea internal standard (data not shown), indicating that it was not cytotoxic under these conditions.

### Cellular uptake of DAPA, and its relationship to diffusion in multicellular membranes

Fluorescence microscopy of V79-171b cells exposed to DAPA while growing on glass coverslips showed a pattern of subcellular distribution consistent with localization in endosomes (Figure 7A). This localization was inhibited by ammonium chloride (50 mM), resulting in diffuse cytoplasmic fluorescence (Figure 7B). Lowering of pH from 7.4 to 6.5 decreased overall fluorescence intensity without preventing localization in vesicles (Figure 7C). A quantitative examination of uptake of [$^3$H]DAPA into V79-171b cells, in single cell suspensions freshly isolated from MM, showed that high ratios of intracellular to extracellular drug were achieved rapidly (*c.* 150-fold by 5 min) at pH 7.2, and that this ratio then increased slowly (Figure 8). Ammonium chloride inhibited this second (slow uptake) component whereas at pH 6.5 uptake was suppressed strongly at all times. The lines in Figure 8 are the fits to an empirical model, in which uptake is driven by binding to saturable and non-saturable intracellular sites (Appendix 2).

The binding parameters from the single cell uptake study (Table 1) were used to represent DAPA binding in V79-171b MM in a flux model based on a reaction-diffusion equation (Appendix 1). In this pre-steady state treatment it was assumed that the MM is a homogeneous compartment through which reaction (binding) and diffusion occur simultaneously. The binding parameters from the single cell experiments at pH 7.2 (Table 1) were used as this value is intermediate between the measured average pH in compartments 1 (pH 7.0) and 2 (pH 7.47). The binding parameters were adjusted for the higher cell density in the MM ($2.9 \times 10^8$ cells ml$^{-1}$, estimated from the cell yield of $5 \times 10^6$ per MM after pronase digestion and

© Cancer Research Campaign 1997

Figure 5 Flux of [$^3$H]DAPA through collagen-coated support membranes without cells (●, 0.5% agar; ■, 1% agar; ▲, 2% agar in compartment 1, $c_{1,0}$ 24 μM) and with V79-171b MM using agar at 0.5% in compartment 1 (○, $c_{1,0}$ 16 μM; □, $c_{1,0}$ 36 μM). DAPA flux through MM was also investigated with 50 mM ammonium chloride in both compartments (△, $c_{1,0}$ 16 μM). Representative curves only are shown for clarity. For the cell-free data the curves are fits for diffusion without binding, with $D_1$ as the fitted parameter. For the MM data, the full reaction–diffusion model (Appendix 1) was used as described in the text the total MM volume calculated from its mean thickness). The MM thickness estimated from the internal standard (urea) flux kinetics was used in each case, and the fitted parameter in the reaction–diffusion model was the $D_{MM}$ for free DAPA. This model gave good fits to the DAPA flux data (Figure 5), yielding mean estimates of $D_{MM}$ for free DAPA of 4.8 ± 0.3 (s.e.m.) × $10^{-7}$ cm$^2$ s$^{-1}$ ($n$ = 9) without ammonium chloride and 5.0 ± 0.7 × $10^{-7}$ cm$^2$ s$^{-1}$ ($n$ = 4) with ammonium chloride. This indicates that the diffusion coefficient of DAPA in the MM (when known binding in cells has been accounted for) is approximately eightfold less than in medium, and that the effect of ammonium chloride on flux can be accounted for by its effect on cellular uptake/binding in single cell suspensions.

### DISCUSSION

The growth of tumour cells as a multicellular layer provides a new in vitro model for the extravascular compartment of tumours. This model can be considered an extension of the use of tumour cell monolayers on microporous supports (Adson et al, 1994, 1995). The latter have been investigated as models for endothelial or epithelial absorption barriers but, being only one cell thick, do not include the heterogeneous microenvironments that arise as a result of limiting transport of nutrients, oxygen and waste products in the extravascular compartment of tumours. In contrast MMs are diffusion-limited structures, as demonstrated by necrosis in regions distant from the nutrient source and the presence of radiobiologically hypoxic cells (Cowan et al, 1996), and by the accumulation of cells with a $G_1$-phase DNA content (data not shown). In these

Figure 6 (A) HPLC radiochromatogram for a sample taken from compartment 2 at 7.5 h during a [$^3$H]DAPA flux experiment, using a V79-171b MM ($c_{1,0}$ 16 μM). (B) The fraction of total radioactivity in compartment 2 due to DAPA for the experiment shown in Figure 3. ○: $c_{1,0}$ 16 μM, without ammonium chloride. □: $c_{1,0}$ 36 μM, without ammonium chloride. △: $c_{1,0}$ 16 μM with 50 mM ammonium chloride.

© Cancer Research Campaign 1997

**Figure 7** Fluorescence microscopy of V79-171b cells, grown on glass coverslips, after incubation with DAPA (50 μM) for 10 min. (**A**) pH 7.4. (**B**) pH 7.4 with ammonium chloride (50 mM). (**C**) pH 6.5. The bar represents 10 μm

**Figure 8** Representative data showing kinetics of uptake of [$^3$H]DAPA by single-cell suspensions (5 × $10^5$ cells $ml^{-1}$), obtained from V79-171b MM, after adding DAPA ($c_0$ 15 μM) at extracellular pH values of 7.6 (▼, ▽), 7.2 (●, ○) and 6.7 (■, □). Closed symbols, without ammonium chloride; open symbols, with ammonium chloride. Mean intracellular concentrations were calculated assuming a water volume of 1 pl per cell. The curves are extended least square fits for the uptake model described in Appendix 2.

**Table 1** Parameters used to fit the flux of [$^3$H]DAPA through V79-171b MM, using the diffusion-with-binding model (Appendix 1)

| | | |
|---|---|---|
| Model parameters | | |
| Length of compartment 1 | | 6.6 mm |
| Thickness of MM[a] | | 235 ± 15 μm |
| Thickness of support membrane | | 30 μm |
| Porosity of support membrane | | 7.3% |
| $D_1$ ($cm^2$ $s^{-1}$) × $10^6$ | | 3.87 ± 0.28 |
| Cell density in MM | | 2.9 × $10^8$ $ml^{-1}$ |
| Transport parameters in the MM | No $NH_4Cl$ | 50 mM $NH_4Cl$ |
| $k_1$[b] ($s^{-1}$) | 24.9 | 24.9 |
| $k_{-1}$[b] ($s^{-1}$) | 1.17 | 1.17 |
| $k_2$[b] ($s^{-1}$ $\mu M^{-1}$) | 0.0018 | 0 |
| $k_{-2}$[b] ($s^{-1}$) | 0.036 | 0 |
| $B_{max}$[b] (μM) | 2382 | 0 |
| Fitted $D_{MM}$ for free DAPA ($cm^2$ $s^{-1}$) × $10^7$ | 4.8 ± 0.3 | 5.0 ± 0.7 |

[a] Determined from urea flux kinetics, using $D_{MM}$ = 1.45 × $10^{-6}$ $cm^2$ $s^{-1}$.
[b] Determined from the single cell uptake kinetics at pH 7.2 (Figure 8). Values for $k_1$ and $B_{max}$ are scaled to the cell density in the MM.

respects MMs closely resemble multicellular spheroids, and other diffusion-limited multicellular systems such as histocultures (Hoffman, 1991) and hollow fibre cultures (Casciari et al, 1994), but offer the important advantage that diffusion of substances can be examined in a direct way by measuring flux across the structure. The MM model is thus ideally suited to examining the extravascular transport characteristics of therapeutic agents, nutrients and macromolecules such as cytokines and growth factors. Initial studies (data not shown) suggest that a variety of rodent and human tumour cell lines can be grown in this manner, and that some cell lines that do not form spheroids efficiently grow well as MM. Thus, this model may find a variety of applications in tumour biology. A similar model has recently been developed by Minchinton et al (1997).

The method for measuring flux across MMs described here (with compartment 1 unstirred after addition of diffusant) is convenient, but will be less sensitive to the impediment imposed by the cellular barrier than if both sides are stirred. Nonetheless, numerical simulation of flux showed that the model is suitably sensitive to $D_{MM}$, especially if the early flux kinetics is examined (Figure 4A). Measured flux under these conditions is very sensitive to any convective disturbance that collapses the concentration gradient in compartment 1, but this problem was conveniently overcome by addition of agar. This also ensured that there was no bulk flow between compartments, which is particularly important in measuring diffusion through the support membrane in the absence of cells. The diffusion equation has no analytical solution under these conditions, but is readily solved using numerical methods.

The use of this method is illustrated by the investigation of urea diffusion. The flux data are well fitted by the simple Fickian diffusion model, both in the presence and absence of the cellular layer (Figure 4B). Good reproducibility is seen between replicate determinations, and the estimated $D_{MM}$ is lower than that in medium (compartment 1) by factors of 11 and 8 for V79-171b and EMT6/Ak MM respectively. The flux of urea in this system is

 © Cancer Research Campaign 1997

clearly faster than that across well-differentiated colon carcinoma (Caco-2) cell monolayers (Adson et al, 1994), although the latter are only *c.* 10 μm in thickness. The flux of this hydrophilic solute appears to be primarily through a paracellular (between cells) pathway in the latter epithelial model, but a contribution from the transcellular (through cells) pathway cannot be ruled out in V79-171b and EMT6/Ak MM.

The very slow flux of DAPA through MM shows that the MM imposes a much more severe impediment to diffusion of this compound. A complication in investigating the diffusion of [$^3$H]DAPA is that the flux is dominated at early times by a minor radioactive and fluorescent hydrolysis product, acridone, that diffuses much more rapidly than the parent compound (Figure 6). This would lead to overestimation of DAPA diffusion rates as measured by autoradiography or fluorescence microscopy, techniques which are often used to examine diffusion in multicellular spheroids or tumour tissue. A key advantage of the MM model is that it allows use of compound-specific analytical methods such as HPLC to identify the diffusing species.

The shape of the flux curve for DAPA could not be described adequately by simple diffusion under steady state conditions (i.e. by assuming a constant effective diffusion coefficient in the MM). The increasing flux with time strongly suggests that the approach to steady state is slow because of a large DAPA-binding capacity in the MM. Such binding might be due to DNA intercalation or other macromolecular binding, but any other reversible entrapment in cells (e.g. active transport, or sequestration of this basic acridine in acidic vesicles driven by pH gradients) would have an analogous effect on flux. The large increase in DAPA flux caused by co-incubation with ammonium chloride (Figure 5) at concentrations known to raise lysosomal pH (Siim et al, 1994) point to sequestration in acidic vesicles as the major impediment to diffusion. Such entrapment in cells was demonstrated by fluorescence microscopy, which showed intense DAPA fluorescence in cytoplasmic vesicles and confirmed that uptake into these vesicles is inhibited by ammonium chloride (Figure 7).

A full spatially distributed model of the effects of lysosomal sequestration on net flux would require detailed information on diffusion coefficients in the extracellular, cytoplasmic and lysosomal compartments, and the transmembrane exchange kinetics and topographical relationships between these compartments. A practical alternative is to approximate the cellular entrapment as reversible binding to sites distributed isotropically within a homogeneous MM. Such lumped parameter modelling of the flux under pre-steady state conditions (Appendix 1) indicated that models of this type could describe the observed flux well. However, within the precision of the data an envelope of solutions was possible, with a wide variety of values for the binding site concentration, reaction (association and dissociation) rate constants, and the diffusion coefficient of the free drug (data not shown). We therefore sought to constrain the model by providing independent estimates of the intracellular binding parameters.

Sequestration of [$^3$H]DAPA by V79-171b cells was fitted well by a simple binding model (Figure 8 and Appendix 2). The intracellular binding parameters determined for single-cell suspensions (after scaling $k_1$ and $B_{max}$ appropriately to allow for the difference in cell density) were applied to the reaction–diffusion model for DAPA flux in V79-171b MM. With the $D_{MM}$ of free DAPA now as the only fitted parameter, this model gave an excellent fit both with and without ammonium chloride (Figure 5). The fitted value of $D_{MM}$ was unaffected by ammonium chloride (i.e. the effect of ammonium chloride is entirely accounted for by its effect on uptake into cells), which gives further support to the model. The value for $D_{MM}$ for free DAPA, eightfold lower than in medium, also appears to be a reasonable estimate for the tissue diffusion coefficient of the free drug as the $D_{MM}$ for urea (this study) and for misonidazole (Cowan et al, 1996) are lower than that in medium by 11- to 12-fold. The high microviscosity of cytoplasm gives diffusion coefficients for (unbound) small ions and non-electrolytes two- to fivefold lower than in water (Mastro and Keith, 1984), but membrane barriers impose an additional two- to fivefold reduction in transcellular diffusion for dyes with MW similar to DAPA (Safranyos et al, 1987).

Although the fit of the DAPA flux to the reaction–diffusion model is impressive, this treatment should be considered illustrative of the potential of the approach, rather than providing definitive transport parameters, as the gradients of extracellular and intracellular pH through the MM have not yet been determined, and the binding rate constants describing cell uptake are very sensitive to pH (Figure 8), as expected for this diprotic base. The flux model used for Figure 5 assumes a single value of 7.2 for the extracellular pH throughout the MM. The fit to the model was less satisfactory when the binding parameters for pH 6.7 or 7.6 were used, indicating that the estimate of pH 7.2 may be reasonable. This is also supported by microelectrode data showing a pH gradient from 7.4 to 7.1 through the viable rim of V79-379A spheroids (Carlsson and Acker, 1988). The other parameters not defined with precision are the intracellular volume fraction in the MM, the fraction of necrotic tissue, and the distribution of thicknesses for each MM. A fully quantitative treatment will require improved (preferably non-destructive) methods for monitoring these parameters.

Although the individual transport parameters for DAPA in V79-171b MM (Table 1) are provisional, together they provide a good empirical model for flux through the MM and thus allow for the first time an estimate of the diffusion time of a cytotoxic drug in the extravascular compartment of tumours based on pre-steady state measurements in an experimental model. The reaction–diffusion model (Appendix 1) was used to calculate diffusion times, assuming a simple planar geometry to provide an approximate estimate. The time required for this DNA intercalator to reach a free drug concentration 90% of that in plasma at a distance of 100 μM into an extravascular compartment is 13 h. Although the pharmacokinetics of this model compound has not been investigated, the required diffusion time will be much longer than the plasma half life, and slow extravascular transport will therefore compromise delivery to cells distant from functional blood vessels.

The impediment to diffusion in tissue imposed by DNA binding of drugs is well recognized, but almost all drugs that bind physically to DNA are also bases, and therefore prone to sequestration in lysosomes. The implications of lysosomal uptake (which is formally analogous to the DNA binding problem) for extravascular drug transport have received less consideration. The use of lysosomotropic bases such as chloroquine may be of interest as a means of overcoming this restriction. We are also investigating prodrug approaches, such as tertiary amine *N*-oxide derivatives of basic DNA intercalators (Wilson et al, 1996), as strategies for enhancing drug delivery. Such prodrugs have the potential for bioreductive release of the active intercalators in hypoxic regions of tumours. The present MM model will be a valuable tool for investigating extravascular transport of these prodrugs and their DNA-binding products.

© Cancer Research Campaign 1997

### ACKNOWLEDGEMENTS

This work was supported by the Health Research Council of New Zealand and the Cancer Society of New Zealand. SJO was the recipient of a scholarship from the Auckland Division of the Cancer Society of New Zealand. We thank Susan Pullen and Kerin Thompson for tissue culture assistance, Dianne Ferry and Dr Frederik Pruijn for assistance with HPLC, Dr Jonathan Zwi for advice with frozen sections, and A/Professor Nicholas Holford for helpful discussions and supply of the MKModel Programme.

### REFERENCES

Adson A, Raub TJ, Burton PS, Barsuhn CL, Hilgers AR, Audus KL and Ho NFH (1994) Quantitative approaches to delineate paracellular diffusion in cultured epithelial monolayers. *J Pharm Sci* **83**: 1529–1536.

Adson A, Burton PS, Raub TJ, Barsuhn CL, Audus KL and Ho NFH (1995) Passive diffusion of weak organic electrolytes across Caco-2 cell monolayers: Uncoupling the contributions of hydrodynamic, transcellular, and paracellular barriers. *J Pharm Sci* **84**: 1197–1204

Baguley BC and Finlay GJ (1995) Pharmacokinetic/cytokinetic principles in the chemotherapy of solid tumours. *Clin Exper Pharmacol Physiol* **22**: 825–828

Carlsson J and Acker H (1988) Relations between pH, oxygen partial pressure and growth in cultured cell spheroids. *Int J Cancer* **42**: 715–720

Casciari JJ, Hollingshead MG, Alley MC, Mayo JG, Malspeis L, Miyauchi S, Grever MR and Weinstein JN (1994) Growth and chemotherapeutic response of cells in a hollow-fiber in vitro solid tumor model. *J Natl Cancer Inst* **86**: 1846–1852

Clauss MA and Jain RK (1990) Interstitial transport of rabbit and sheep antibodies in normal and neoplastic tissues. *Cancer Res* **50**: 3487–3492

Cowan DSM, Hicks KO and Wilson WR (1996) Multicellular membranes as an in vitro model for extravascular diffusion in tumours. *Br J Cancer* **73** (suppl. XXVII) S28–S31

Crank J (1975) *The Mathematics of Diffusion*, 2nd edn. Clarendon Press: Oxford

Cussler EL (1984) *Diffusion, Mass Transfer in Fluid Systems*. Cambridge University Press: New York

Durand RE (1989) Distribution and activity of antineoplastic drugs in a tumor model. *J Natl Cancer Inst* **81**: 146–152

Durand RE (1990) Slow penetration of anthracyclines into spheroids and tumors a therapeutic advantage? *Cancer Chemother Pharmacol* **26**: 198–204

Durand RE and Olive PL (1992) Evaluation of bioreductive drugs in multicell spheroids. *Int J Radiat Oncol Biol Phys* **22**: 689–692

Groebe K, Erz S and Mueller-Klieser W (1994) Glucose diffusion coefficients determined from concentration profiles in EMT6 tumor spheroids incubated with radioactively labeled L-glucose. *Adv Exper Biol Med* **361**: 619–625

Hoffman RM (1991) Three-dimensional histoculture: origins and applications in cancer research. *Cancer Cells* **3**: 86–92

Jain RK (1987) Transport of molecules in the tumor interstitium: A review. *Cancer Res* **47**: 3039–3051

Jain RK and Baxter LT (1988) Mechanisms of heterogeneous distribution of monoclonal antibodies and other macromolecules in tumors: significance of elevated interstitial pressure. *Cancer Res* **48**: 7022–7032

Jain RK and Baxter LT (1993) Extravasation and interstitial transport in tumors. In *Biological Barriers to Protein Delivery*, Audus KL and Raub TJ (eds), pp. 441–465. Plenum Press: New York

Kerr DJ and Kaye SB (1987) Aspects of cytotoxic drug penetration with particular reference to anthracyclines. *Cancer Chemother Pharmacol* **19**: 1–5

Kwok TT and Twentyman PR (1987) Use of a tritiated thymidine suicide technique in the study of the cytotoxic drug response of cells located at different depths within multicellular spheroids. *Br J Cancer* **55**: 367–374

Ledochowski A, Ledochowski Z, Peryt J and Wojciechoswska H (1964) Research of tumour-inhibiting compounds. XXIV. Some N-9 derivatives of 9-aminoacridine. *Roczniki Chemii* **38**: 1111–1114

Mastro AM and Keith AD (1984) Diffusion in the aqueous compartment. *J Cell Biol* **99**: 180–187

Minchinton AI, Wendt KR, Clow KA and Fryer KH (1997) Multilayers of cells growing on a permable support – an in vitro tumour model. *Acta Oncologica* **36**: 13–16

Nederman T, Carlsson J and Malmqvist M (1981) Penetration of substances into tumor tissue – A methodological study on cellular spheroids. *In Vitro*, **17**: 290–298

Olive PL (1986) Patterns of mutagen binding and penetration in multicell spheroids. *Environmen Mutagen* **8**: 705–715

Peck C, Beal SL, Sheiner LB and Nichols AI (1984) Extended least squares nonlinear regression: A possible solution to the choice of weights problem in analysis of individual pharmacokinetic data. *J Pharmacokin Biopharm* **12**: 545–558

Roberts PB, Denny WA, Wakelin LPG, Anderson RF and Wilson WR (1990) Radiosensitization of mammalian cells in vitro by nitroacridines. *Radiat Res* **123**: 153–164

Safranyos RGA, Caveney S, Miller JG and Petersen, NO (1987) Relative roles of gap junction channels and cytoplasm in cell-to-cell diffusion of fluorescent tracers. *Proc Natl Acad Sci USA* **84**: 2272–2276

Siim BG, Denny WA and Wilson, WR (1994) Does DNA targeting affect the cytotoxicity and cell uptake of basic nitroquinoline bioreductive drugs? *Int J Radiat Oncol Biol Phys* **29**: 311–315

Sutherland RM (1988) Cell and environment interactions in tumor microregions: the multicell spheroid model. *Science* **240**: 177–184

Vaupel P, Kallinowski F and Okunieff, P (1989) Blood flow, oxygen and nutrient supply and metabolic microenvironment of human tumours: A review. *Cancer Res* **49**: 6449–6465

Vistica DL (1979) Cytotoxicity as an indicator for transport mechanism. Evidence that melphalan is transported by two leucine-preferring carrier systems in the L1210 murine leukemia cell. *Biochim Biophys Acta* **550**: 309–317

Wilson WR and Denny WA (1992) DNA-binding nitroheterocycles as hypoxia-selective cytotoxins. In *Radiation Research, a Twentieth-Century Perspective*, vol. 2, Dewey WC, Edington M, Fry RJM, Hall EJ and Whitmore GF (eds), pp. 796–801. Academic Press: San Diego

Wilson WR, Whitmore GF and Hill RP (1981) Activity of 4′-(9-acridinylamino) methanesulphon-*m*-anisidide (*m*-AMSA) against Chinese hamster cells in multicellular spheroids. *Cancer Res* **41**: 2817–2822

Wilson WR, Denny WA, Stewart GM, Fenn A and Probert JC (1986) Reductive metabolism and hypoxia-selective toxicity of nitracrine. *Int J Radiat Oncol Biol Phys* **12**: 1235–1238

Wilson WR, Denny WA, Pullen SM, Thompson KM, Li AE, Patterson LH and Lee HH (1996) Tertiary amine N-oxides as bioreductive drugs: DACA *N*-oxide, nitracrine *N*-oxide and AQ4N. *Br J Cancer* **74** (suppl. XXVII): S43–S47

### APPENDIX 1: DIFFUSION WITH REACTION IN MULTICELLULAR MEMBRANES

Drug diffusion in each of three or four compartments in series (compartment 1, MM if present, support membrane and compartment 2) was modelled assuming each to be a homogeneous phase in which binding to saturable and non-saturable sites can occur, and that the bound drug is immobile. The equations for this reaction–diffusion model are:

$$\frac{\partial c_f}{\partial t} = D_f \frac{\partial^2 c_f}{\partial x^2} - k_1 c_f + k_{-1} c_{b1} - k_2 c_f (B_{max} - c_{b2}) + k_{-2} c_{b2}$$

$$\frac{\partial c_{b1}}{\partial t} = k_1 c_f - k_{-1} c_{b1}$$

$$\frac{\partial c_{b2}}{\partial t} = k_2 c_f (B_{max} - c_{b2}) - k_{-2} c_{b2}$$

where $c_f$, $c_{b1}$, $c_{b2}$ are the concentrations of free drug, drug bound to the non-saturable sites and to the saturable sites respectively, at position $x$ and time $t$; $D_f$ is the diffusion coefficient of the free drug; $k_1$ and $k_{-1}$ are the forward (association) and reverse (dissociation) rate constants, respectively, for non-saturable binding; $k_2$ and $k_{-2}$ are the forward and reverse rate constants, respectively, for saturable binding; $B_{max}$ is the total concentration of saturable binding sites. In the absence of binding (i.e. $k_1 = k_{-1} = k_2 = k_{-2} = 0$), the reaction–diffusion equations reduce to Fick's second law. The boundary conditions are for zero flux at the upper surface of compartment 1 ($x = 0$) and the lower surface of compartment 2 ($x = B$),

© Cancer Research Campaign 1997

$$\frac{\partial c_f}{\partial x} = 0, \text{ at } x = 0, x = B, t \geq 0$$

and initial conditions $c_f\,(x,0) = c_{1,0}$ in compartment 1, $c_f(x,0) = 0$ otherwise, and $c_{b1}\,(x,0) = c_{b2}(x,0) = 0$ in all compartments.

The parameters take on different values in each compartment. The values of the binding parameters for the MM compartment were obtained experimentally from the kinetics of binding to single cells (Appendix 2). In both the MM and single-cell systems, the binding sites are intracellular but are averaged over the whole volume. The values of $k_1$ (proportional to the non-saturable binding site concentration) and $B_{max}$ obtained from the single cell data were therefore multiplied by the ratio of cell densities in the MM and single cell system (see Results).

The system of partial differential equations for diffusion through all compartments was solved numerically using a finite difference method in the NAG Library routine DO3PBF, on a VAX 11/750 minicomputer. The predicted compartment 2 concentration–time curve was fitted to the data using the diffusion coefficient for free drug in one of the compartments (i.e. $D_1$, $D_{MM}$ or $D_s$) as the fitted parameter, with minimization of the sum of squared deviations as the fitting criterion. $D_2$ was set at 1000 cm$^2$ s$^{-1}$ to model continuous stirring in compartment 2. The software was validated by calculating the concentration–time profile at a distance of 0.5 cm from a plane boundary between two unstirred compartments with lengths of 0.5 cm ($D$ = 10$^{-5}$ cm$^2$ s$^{-1}$; $c(x,0)$ = 10 µM for $0 \leq x < 0.5$ and $c(x,0)=0$ for $0.5 \leq x \leq 1$). The calculated concentrations were within 1% of the analytical solution (Crank, 1975) at all times after 10 min.

### APPENDIX 2: KINETICS OF DRUG UPTAKE MODELLED AS INTRACELLULAR BINDING

The uptake of DAPA by single cells was also treated as a homogeneous binding problem to make the mathematical formalism compatible with the reaction–diffusion model above. In this approach cellular uptake was assumed to be due to reversible intracellular binding alone (which, using a continuum approximation, is mathematically equivalent to reversible sequestration into subcellular vesicles). In this case the extracellular drug concentration ($c_e$) can be considered a measure of the concentration ($c_f$) of the free drug, and the total intracellular concentration, $c_i$, is equal to total intracellular bound and free drug.

Experiments examining $c_i$ as a function of $c_e$ (data not shown) were compatible with a high-affinity saturable binding mode plus a non-saturable low-affinity mode. A model consisting of two classes of intracellular binding sites was also necessary to adequately describe the kinetics of DAPA binding by single V79-171b cells (Figure 8).

The equations describing the cellular binding model are:

$$\frac{dc_{b1}}{dt} = k_1 c_f - k_{-1} c_{b1}$$

$$\frac{dc_{b2}}{dt} = k_2 c_f\,(B_{max} - c_{b2}) - k_{-2} c_{b2}$$

Cellular binding caused loss of drug from the medium and hence $c_f$ decreased according to:

$$c_f = c_o - c_{b1} - c_{b2}$$

The initial conditions are $c_f = c_o$, the initial concentration of drug in the extracellular medium, and $c_{b1} = c_{b2} = 0$.

The model predictions for $c_e$ and $c_i$ were fitted to the measured values from the uptake experiments using the programme MKModel, with $k_1$, $k_{-1}$, $k_2$, $k_{-2}$, and $B_{max}$ as fitted parameters. The differential equation solver in MKModel uses a 4th order Range–Kutta method with minimization of the extended least squares function (Peck et al, 1984) as fitting criterion. The data were fitted to pairs of curves at the same pH, assuming that the saturable binding component was eliminated ($k_2 = k_{-2} = B_{max} = 0$) in the presence of ammonium chloride.

© Cancer Research Campaign 1997